# The impact of videogames on the mood of amateur youth players during consecutive games

**DOI:** 10.3389/fspor.2023.1309918

**Published:** 2023-12-05

**Authors:** Adrián Mateo-Orcajada, Raquel Vaquero-Cristóbal, Ana María Gallardo-Guerrero, Lucía Abenza-Cano

**Affiliations:** ^1^ Facultad de Deporte, UCAM Universidad Católica de Murcia, Murcia, Spain; ^2^ Department of Physical Activity and Sport, Faculty of Sport Sciences, University of Murcia, Murcia, Spain

**Keywords:** amateur, competitive games, esports, moods, psychology, social impact

## Abstract

**Introduction:**

Esports have experienced tremendous growth in recent years. In the scientific field, previous research has shown the determining role of psychology in competitive performance, but little is known about the factors that may be more determinant. In addition, in the amateur field, where fun and enjoyment are the most important factors, it has been observed that players can see their psychological state altered due to different factors, but it is not known if the outcome of the game (win or lose) can be influential. For this reason, the aim of the present investigation was to analyze changes in players' mood between three consecutive games as a function of the outcome of each game.

**Methods:**

A total of 14 amateur players participated in the research, all of them with previous experience and being regular League of Legends players. The participants completed the POMS questionnaire before the start of each game and the outcome of each game was recorded at the end.

**Results:**

The results showed that no significant pre-game differences were found in any of the games, regardless of winning or losing. Significant differences were found in the pre-game mood between the first and second game, according to the outcome of the first game, and between the second and third game, according to the outcome of the second game. Between the first and second games, there was a significant increase in depression (*p* = 0.038) and anger (*p* = 0.003) when the first game was lost; and between the second and third games, there was a decrease in tension (*p* = 0.003) and anger (*p* = 0.022) when the second game was won. In addition, it should be noted that fatigue increased significantly after each game, regardless of the outcome, and with respect to the change in mood, this was more noticeable when the first game was lost and the second was won, as significant changes were observed in tension (*p* = 0.028), depression (*p* = 0.030) and anger (*p* = 0.006).

**Conclusion:**

Pre-match mood does not influence post-match performance, but mood changes do occur between successive matches depending on the outcome of the match.

## 1. Introduction

Esports, considered online video game competitions, have experienced a great growth and development in recent decades, leading to a massive increase in the number of players, spectators, and competitions worldwide (1–3). Competition is the most relevant element of Esports, with large economic investments being made and large-capacity facilities being used to host the events (4, 5). Due to its relevance, scientific research has been carried out in recent years to discover the most determinant variables in the performance of players during competitions (6–9), finding that environmental and personal variables are determinant. With respect to environmental variables, the light condition has been shown to be especially relevant, since the excessive screen time (exceeding 2 or 3 h of continuous play) to which esports players are subjected produces cognitive fatigue with pupillary constriction, decreasing cognitive performance (10), as well as eye fatigue (11). Regarding personal variables, sleep, nutrition, or psychological state, have been the most studied in the competition environment. Thus, previous research have shown that the performance of the players was diminished by sleep disturbances that affect the rest of the players, being furthermore likely to present sleep disorders due to the unique situations and conditions that characterize them (12) and that although esports players maintain a regular sleep pattern, most do not reach the minimum recommended sleep guidelines, with the best performing players being those who slept more, spent more time in deep sleep and less time in light sleep, showed lower non-REM respiration rates per minute, and earlier sleep offset (8); that nutrition and supplementation also seemed relevant, with performance being higher when nutrition was adequate, but more scientific evidence is needed to corroborate this (9); and that the psychological state of players seemed to influence competitive performance (6), and was also related to changes in certain physiological parameters such as heart rate (13).

So much so that the existing relationship between performance and different health parameters leads esports players to be at biopsychosocial risk, with their behavior being characterized by excessive caffeine supplementation, physiological arousal, injury, pain, stress, maladaptive coping, cognitive fatigue, game addiction or bullying, which affects their well-being (14). In addition to these unhealthy behaviors, there is another personal variable of great relevance, such as the excessive workload to which players are subjected (15). As esports are still in the development phase, not much information is available about workload control, subjecting players to long hours of training and competition (15). In this regard, heart rate, and more specifically heart rate variability, have started to be used in esports due to its potential to monitor player self-regulation (16). Thus, recent research has shown changes in HRV time-domain variables during games, as well as that the mean standard deviation of RR intervals was lower in the winning team than in the losing team, with some areas of analogous change in heart rate variability also existing in players at the end of matches (17); in addition, heart rate did not change when comparing games won and lost, but significant differences were found in heart rate according to the action performed, with those that directly involved the player and favored the team increasing hear rate the most (7). However, studies on workload, and more specifically on heart rate are limited in esports, and although they are used to understand the response to stress and the workload, there is a lack of theoretical evidence and methodological foundations to draw conclusions (16).

Of all esports, most of the previous research conducted so far has been developed on League of Legends (LOL), since it has a large number of professional and amateur players worldwide, being the most professionalized video game (18). LOL belongs to the multiplayer online battle arena (MOBA) genre, characterized by competitive, fast-paced games with teams generally consisting of five players trying to destroy the rival base (19). This form of gameplay gives rise to numerous interactions between the players in one's own team and those of the rival team (20, 21), which could lead to modifications in the psychological and physiological state of the players depending on the evolution of the game (6, 22). Not surprisingly, research conducted on professional LOL players has tried to establish the relationship between individual and collective performance in competition with psychological and technical-tactical performance variables (22, 23). More specifically, it has been found that in case of defeat in competition, changes in the mood of professional players occur, characterized by a significant decrease in vigor, and a significant increase in tension, depression, anger, and fatigue after the game (6). In addition, the effectiveness of psychological interventions to help players regulate their emotions and remain calm before and during games has been demonstrated (23, 24); as well as the influence of internal communication between players on the same team on individual and collective performance (25, 26).

However, although most of the research in LOL has analyzed psychological variables, scientific evidence is scarce. Thus it has been observed that mood changes during the game (6), but nothing is known about how mood behaves during successive games, nor whether the outcome of each game influences the mood with which the next game is approached. This is due to the fact that the majority of this research has analyzed what happens in LOL in isolation, considering the games as a single event (6). However, one of the great attractions of LOL for spectators are the final stages of the championship in which the teams face each other in a best of three or five consecutive games (Bo3 or Bo5), with the winner being the one that defeats the other team two or three times, respectively (27). Therefore, this is one of the main limitations observed in previous scientific literature investigating single games or regular leagues in which the games are separated by several days of rest (28).

This gap in the scientific literature on esports is important because it is known that in traditional sports, consecutive matches influence certain performance variables. Thus, it has been observed that in volleyball, consecutive matches affect heart rate variability (29); while in basketball, consecutive matches affect kinematic demands (relative distance, high intensity running, peak speed, deceleration) (30), as well as physical condition and psychological state (31). In esports, studies on the influence of congestion resulting from consecutive matches are practically nonexistent, but it has been observed that weeks with a higher number of games lead to poorer sleep quality for players (32). The absence of research on game congestion in esports becomes even more relevant when considering that in esports, the psychological component is more predominant than the physical one, and alterations of the psychological state are associated with disruptive behaviors during games (33, 34). This type of behaviours are frequent in LOL games, as in 70% of games players are confronted with annoying situations, including disruptive behaviour towards their teammates or opponents (35, 36). This is especially important because, beyond the professional competitive environment, where players must perform to achieve the aims set by the team, in the amateur environment more and more players spend their free time playing LOL (37). This allows numerous players to share playful gaming experiences, with friends or strangers, becoming one of the main relevant form of digital entertainment (38–40). Therefore, the benefits of LOL as a video game could be undermined by a chain of negative outcomes in successive games, making players more vulnerable to manifest disruptive behaviors that would make the gaming experience negative.

Given that negative gaming experiences with other players keep players in a negative mood hours after the end of games (33, 41), are one of the main reasons to stop playing MOBAs (42, 43), and knowing that the mood prior to the game can predispose to the occurrence of this type of behaviors during games (33), it is necessary to know whether the results obtained in consecutive LOL games can attenuate or worsen the mood with which players face the next game. Even more so when considering that consecutive games of esports negatively affect the players' rest (32), so it could also affect mood. There is no previous research in this regard, and this study would make it possible to offer recommendations to players of this video game in particular, and esports in general, so that they are not so affected by the results of the games and do not stop playing it. For this reason, the aim of the present research was to analyze changes in the mood of amateur LOL players between three consecutive games as a function of the outcome of each game.

According to previous research, the following research hypothesis is proposed: H1) the mood of amateur LOL players will be affected by the outcome of each game, with the mood worsening to a greater extent with defeats occurring consecutively compared to defeats following a win.

## 2. Materials and methods

### 2.1. Design

The study design was cross-sectional, with non-probability convenience sampling. All the study participants signed the informed consent form prior to data collection, and were informed of the objectives of the study, as well as the processing of the data and the confidentiality of their treatment. Both the design and the development of the manuscript followed the STROBE statement (44), and the Institutional Ethical Committee reviewed and authorized the protocol designed for data collection, according to the Code from the World Medical Association (code: CE112002). The present research also follow the guidelines of the Helsinki declaration (to meet satisfactory ethical standards during scientific research).

### 2.2. Participants

The study sample size was calculated using Rstudio 3.15.0 (Rstudio Inc., USA). The significance level was set at *α* = 0.05. The standard deviation (SD) was established based on previous studies that examined mood measures, tension (mean SD = 6.33), depression (mean SD = 10.78), anger (mean SD = 9.20), vigor (mean SD = 8.27), and fatigue (mean SD = 5.54) (6) in League of Legends games. Assuming an estimated error (d) of 3.31 for tension, 5.64 for depression, 4.82 for anger, 4.33 for vigor, and 2.90 for fatigue, the minimum sample needed for this study was 12 players.

The final sample consisted of 14 amateur League of Legends players (mean age: 22.36 ± 3.15; mean League of Legends experience: 5.14 ± 1.61 years) who voluntarily participated in the study. From each participant, three games were analyzed, so a total of 42 games were included in the analysis of the present research. The selection of the sample was carried out by non-probabilistic convenience sampling, selecting all possible subjects who had access and met the inclusion criteria: (1) have at least two years of experience in League of Legends; (2) be a regular player, referring to having played at least one game in the last week; (3) not being a professional player; and (4) and have one of the qualifying ranks (iron, bronze, silver, gold, platinum, diamond, master, grandmaster, or challenger) in competitive League of Legends games.

The small size of the final sample (*n* = 14) is due to the fact that most of the players contacted (initial *n* = 63) did not meet any of the inclusion criteria. More specifically, the players did not meet criteria 2 (be a regular player) and 4 (have one of the qualifying ranks). This is because previous research has shown that most esports players, and more specifically LOL players, are sporadic players, who play isolated games and can spend up to 100 days to return to the game (45). This made it difficult to include players, as many of the players initially contacted did not have a qualifying rank as they had not played games for months.

### 2.3. Instruments

For the League of Legends data collection, all the players used similar computers with the following characteristics: Asus Intel Core i7 8700 k 3.7 Ghz (Intel Corporation, United States), Venom N10 liquid cooling (Netway, Spain), Asus GeForce GTX 1060 6 GB DDRS graphics card (ASUSTeK Computer, Taiwan).

To obtain the psychological score, the abbreviated Spanish form of the Profile of Mood States (POMS) questionnaire was used (46, 47), which had an adequate internal consistency, as shown by the Cronbach's alpha values between 0.70 and 0.83 for the five dimensions analyzed (47). This questionnaire consists of 29 items that allow the evaluation of five mood-related factors; four negative (tension, depression, anger, and fatigue) and one positive (vigor), using a four-point Likert scale for its scoring (0 = not at all; 1 = a little; 2 = moderately; 3 = quite a lot; 4 = very much). To determine the value corresponding to each of the five variables (tension, depression, anger, fatigue, and vigor), the value given, from 0 to 4, to each question of that dimension was added up. To obtain the value for each game, the sum of the values obtained by all the players in each dimension was averaged, following the methodology by Vega-Marcos et al. (48).

### 2.4. Procedure

In the present study, all the participants played three games, separated by a 10-minute rest period, with this amount of time being equal to that provided between successive competitive games. Before starting each of the games and selecting the champions with whom the game would be played, the participants self-completed the POMS questionnaire. The values obtained from the POMS questionnaire were used to determine the pre-game moods. Thus, the differences between the mood states prior to the different games played were used to establish the changes produced by the outcome of the games in the psychological state of the players.

The players entered a competitive game, chose their game position (top laner, jungler, mid laner, adc or support) and the champion with whom they would play the game. The LOL matchmaking system, matching players from both teams by ranking, equalizing the level of both teams, allowed the games to be evenly matched, preventing this from conditioning the player's mood before the start of the game (49, 50). The different games were played, during which a record was made of the team performance (game win or lose). After the end of the game, a 10-minute rest period was allowed, and the player returned to fill in the POMS before the next game began. This process was repeated for the three games played by each player.

As these were amateur players, each was allowed to choose the playing position they preferred and in which they felt most comfortable in each game, as this has not been shown to be relevant to the playing experience in previous research (51). In addition, the use of any champion was not limited. The researchers did not give any indication to the player about the composition of the teams, or the champions selected, trying to influence the player's performance and mood as little as possible. Similarly, at the end of the game, there was no interaction with the player so as not to influence his mood, and he was only warned after 10 min to start the next game. Playing with friends was not allowed, as this could affect the player's mood during the game, so he was asked to enter the game alone with four other unknown teammates. The environmental conditions in which the games were played were similar in all games and for all players, with a stable ambient temperature; with no one who could disturb or distract the players during the game, since they were in a separate room; and with ideal light and screen brightness conditions for playing.

### 2.5. Statistical analysis

The distribution of the data was initially evaluated using the Shapiro-Wilk normality test. As the variables followed a normal distribution, a statistical analysis based on parametric tests was performed. Descriptive statistics were used to find the mean values and standard deviation (M ± SD). A student's *t*-test was conducted to determine differences in players' pregame tension, depression, anger, vigor, and fatigue between games that ended in victory or defeat. A two-way ANOVA with repeated measures was carried out to analyze pre-post game mood change according to the result of the previous and present game. An analysis of the differences in the change produced in the mood prior to the game as a function of the results obtained in the first and second game. Partial eta squared was used to calculate the effect size (ES) and was defined as small: ES ≥ 0.10; moderate: ES ≥ 0.30; large: ≥1.2; or very large: ES ≥ 2.0, with an error of *p* < 0.05 (52). A value of *p* < 0.05 was set to determine statistical significance. The statistical analysis was performed with the SPSS statistical package (v. 25.0; SPSS Inc., IL).

## 3. Results

Table 1 shows the differences in the pre-game mood of the players between the games that ended in victory and defeat. It should be noted that no significant differences were found in the pre-game mood in any of the games played, regardless of the subsequent outcome of the game. For the tension variable, there were no significant pre-game differences in any of the games, regardless of winning or losing (*p* = 0.154–0.618), with the effect size being small in all games; in depression something similar occurred, with no significant differences in any of the games (*p* = 0.351–0.898), with a small effect size; in anger there were also no differences (*p* = 0.114–0.897) and the effect size remained small; as well as in vigor (*p* = 0.155–0.399) and fatigue (*p* = 0.138–0.584) where no differences were found and the effect sizes were small.

**Table 1:** Differences in pre-game mood state between games won and lost.

	Game	Pre-game value (game won)	Pre-game value (game lost)	*t*	*p*	ES
Tension	Game 1	7.50 ± 5.01	8.88 ± 4.94	0.262	0.618	0.021
	Game 2	9.25 ± 5.12	6.83 ± 1.60	1.224	0.290	0.093
	Game 3	8.14 ± 4.26	5.29 ± 2.56	2.312	0.154	0.162
Depression	Game 1	2.17 ± 2.56	2.00 ± 2.20	0.017	0.898	0.001
	Game 2	4.75 ± 5.39	2.50 ± 1.87	0.942	0.351	0.073
	Game 3	2.71 ± 3.64	3.29 ± 3.90	0.080	0.782	0.007
Anger	Game 1	4.33 ± 1.86	7.13 ± 3.64	2.909	0.114	0.195
	Game 2	11.13 ± 9.57	10.50 ± 7.40	0.018	0.897	0.001
	Game 3	9.00 ± 7.57	6.71 ± 4.15	0.490	0.497	0.039
Vigor	Game 1	10.00 ± 3.52	12.12 ± 5.08	0.765	0.399	0.060
	Game 2	9.75 ± 3.54	12.00 ± 4.10	1.214	0.292	0.092
	Game 3	10.86 ± 3.24	8.00 ± 3.79	2.303	0.155	0.161
Fatigue	Game 1	5.17 ± 5.49	3.62 ± 4.75	0.317	0.584	0.026
	Game 2	5.63 ± 5.40	1.83 ± 2.48	2.520	0.138	0.174
	Game 3	4.71 ± 3.40	6.29 ± 6.45	0.325	0.579	0.026

Table 2 shows the change in pre-game mood between the first and second game, according to the outcome of the first game, as well as between the second and third game, according to the outcome of the second game. The results obtained showed significant differences in depression (*p* = 0.038) and anger (*p* = 0.003) between the first and second game, with a significant increase in the score of both variables before starting the second game when the first game was lost. Differences between the second and third games were significant in tension (*p* = 0.003) and anger (*p* = 0.022) when the second game was won, with a significant decrease in both variables.

**Table 2:** Differences in pre-game mood state between consecutive games depending on the outcome of the game.

	Game 1 result	Pre-game 1 (I)	Pre-game 2 (J)	Diff. I-J	95% CI diff.	*p*	ES (η2)	Game 2 result	Pre-game 2 (I)	Pre-game 3 (J)	Diff. I-J	95% CI diff.	*p*	ES (η2)
Tension	Win	7.50 ± 5.01	6.83 ± 3.49	0.667	−1.676; 3.009	0.547	0.031	Win	9.25 ± 5.12	6.50 ± 4.04	2.750	1.172; 4.328	0.003	0.546
	Lose	8.88 ± 4.94	9.25 ± 4.40	−0.375	−2.403; 1.653	0.694	0.013	Lose	6.83 ± 1.60	7.00 ± 3.52	−0.167	−1.988; 1.655	0.845	0.003
Depression	Win	2.17 ± 2.56	1.17 ± 1.60	1.000	−3.056; 5.056	0.601	0.023	Win	4.75 ± 5.39	2.25 ± 3.58	2.500	−1.705; 6.705	0.220	0.123
	Lose	2.00 ± 2.20	5.75 ± 4.68	−3.750	−7.263; −0.237	0.038	0.311	Lose	2.50 ± 1.87	4.00 ± 3.80	−1.500	−6.355; 3.355	0.514	0.036
Anger	Win	4.33 ± 1.86	3.67 ± 1.211	0.667	−5.411; 6.744	0.815	0.005	Win	11.13 ± 9.57	4.50 ± 2.73	6.625	1.130; 12.120	0.022	0.365
	Lose	7.13 ± 3.64	16.25 ± 7.23	−9.125	−14.388; −3.862	0.003	0.543	Lose	10.50 ± 7.40	12.33 ± 6.38	−1.833	−8.179; 4.512	0.541	0.032
Vigor	Win	10.00 ± 3.52	9.50 ± 4.23	0.500	−1.701; 2.701	0.630	0.020	Win	9.75 ± 3.54	8.75 ± 4.13	1.000	−1.005; 3.005	0.299	0.090
	Lose	12.12 ± 5.08	11.62 ± 3.46	0.500	−1.406; 2.406	0.578	0.026	Lose	12.00 ± 4.10	10.33 ± 3.14	1.667	−0.649; 3.982	0.143	0.170
Fatigue	Win	5.17 ± 5.49	2.33 ± 1.86	2.833	−1.249; 6.915	0.156	0.160	Win	5.63 ± 5.40	6.50 ± 6.37	−0.875	−3.810; 2.060	0.528	0.034
	Lose	3.62 ± 4.75	5.25 ± 5.83	−1.625	−5.160; 1.910	0.336	0.077	Lose	1.83 ± 2.48	4.17 ± 2.23	−2.333	−5.722; 1.056	0.159	0.158

Game 1 is the first game played and game 2 is the second game played.

**Figure 1:**
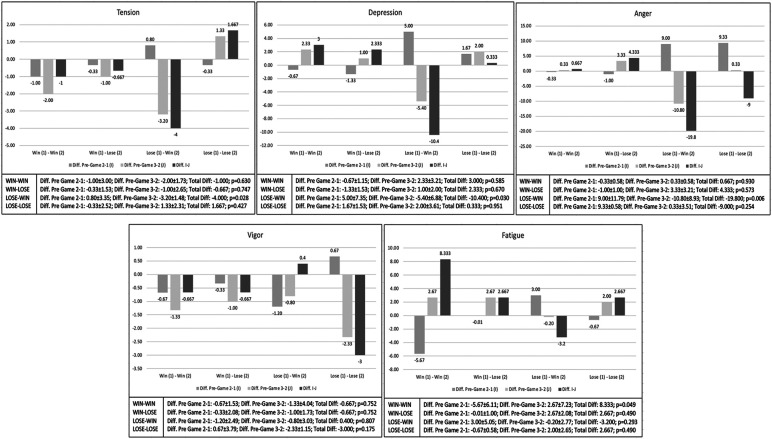
Analysis of the change in mood prior to the game based on the results obtained in the first (1) and second (2) games.

Figure 1 shows the differences in the change of mood pre-post game between the first and second game, according to the outcome of both games. After winning the first and second game, there were only significant differences in fatigue (*p* = 0.049). This is because after the second game there was a considerable increase in this variable, making the change between the two games significant (effect size: 0.334; 95% CI: 0.048; 16.618). In tension (*p* = 0.630; effect size: 0.024; 95% CI: −5.481; 3.481), depression (*p* = 0.585; effect size: 0.031; 95% CI: −8.850; 14.850), anger (*p* = 0.930; effect size: 0. 001; 95% CI: −15.918; 17.252) and vigor (*p* = 0.752; effect size: 0.010; 95% CI: −5.243; 3.909) there were no significant differences after achieving two victories. When the first game was won, but the second game was lost, no significant differences were found in any of the mood states [(tension: *p* = 0.747; effect size: 0.011; 95% CI: 5.148; 3.814); (depression: *p* = 0.670; effect size: 0.019; 95% CI: −9.517; 14.183); (anger: *p* = 0.573; effect size: 0.033; 95% CI: −12.252; 20.918); (vigor: *p* = 0.752; effect size: 0.010; 95% CI: −5.243; 3.909); (fatigue: *p* = 0.490); effect size: 0.049; 95% CI: −5.618; 10.952)]. When the first game was lost but the second game was won, the differences in change were significant in tension (*p* = 0.028; effect size: 0.397; 95% CI: −7.471; −0.529), depression (*p* = 0.030; effect size: 0.389; 95% CI: −19.579; −1.221) and anger (*p* = 0.006; effect size: 0.541; 95% CI: −32.647; −6.953), but not in vigor (*p* = 0.807; effect size: 0.006; 95% CI: −3.145; 3.945) or fatigue (*p* = 0.293; effect size: 0.110; 95% CI: −9.618; 3.218). However, when both games were lost, there was no significant change in either mood [(tension: *p* = 0.427; effect size: 0.064; 95% CI: −2.814; 6.148); (depression: *p* = 0.951; effect size: 0.001; 95% CI: −11.517; 12.183); (anger: *p* = 0.254; effect size: 0.128; 95% CI: −25.585; 7.585); (vigor: *p* = 0.175; effect size: 0.176; 95% CI: −7.576; 1.576); (fatigue: *p* = 0.490); effect size: 0.049; 95% CI: −5.618; 10.952)].

## 4. Discussion

The aim of the present research was to analyze changes in the mood of amateur LOL players between three consecutive games as a function of the outcome of each game. It is important to note that no significant differences were found in the mood prior to the game, regardless of whether the game ended in victory or defeat. This shows that the initial situation of the players was the same in each of the games, and that it was not determinant for the final result of the game. These results are similar to those found in previous research with professional LOL players, in which the pre-game mood of the players was not found to significantly influence whether they won or lost the game (6). A possible explanation for this is that, similar to sports such as soccer, in which the actions in the last minutes of the game are more determinant for victory (53), and the final result does not depend on the players' pre-game mood (54), in LOL the game becomes more important as time progresses, since the players' respawn times are higher and the objectives being fought for become more relevant (18). Therefore, the mood of the players may change throughout the game as the game progresses, as has been seen in traditional modalities (55), and the mood in the final moments of the game may be very important for the final outcome of the competition, to the detriment of the initial mood.

Regarding the evolution of the players’ mood with the game result, there was a significant increase in depression and anger before the start of the second game when the players had lost the first game, while there was a significant decrease in anger and tension when they won the second game. These results are particularly novel as no similar research is known in either the professional or amateur field. Previous research in Esports has not analyzed changes between successive games but has shown changes in players' moods based on the outcome of a single game, with decreases in anger after winning the game and increases in depression, anger, fatigue, and confusion after losing the game (6). Therefore, the results of this study confirm that the mood of amateur players undergoes changes depending on the outcome of the game. However, it is of vital importance to consider the outcome of the previous game when analyzing the change in mood in the subsequent games, as in the second game in the present study, the players started with a higher anger and tension value than the basal state before starting the first game.

To understand the importance of the previous result on the mood of the players in the following game, the pre-post game changes produced on the psychological state of the players were analyzed according to the result of the previous and present game. Thus, the results showed that after winning both games, fatigue increased significantly; after winning the first game and losing the second one, no significant differences were found in either mood; after losing the first game and winning the second, there was a significant decrease in tension, depression and anger at the end of the second game; and after losing both games, no significant differences were found in either mood. In the field of sports, previous research in swimming showed that significant changes in tension, anger, fatigue, and confusion were found on several consecutive days of competition, regardless of race results (56). These results could be due to the fact that competition is a stressful event for professional and non-professional athletes, affecting the psychological state of the competitors, among others (57). The present study is the first to analyze the changes in the mood of amateur esports players between games, showing that fatigue increases progressively, even when winning, so rest between consecutive games is essential, and that tension, depression and anger must be controlled after defeat.

In the field of esports only one previous research had analyzed the influence of a congested period of games on the health of players (32). This study showed that in weeks with a high number of games, the quality of players' sleep was impaired (32). The present research provides new and relevant information in this area, following the line of previous research in sports such as basketball in which it was observed that consecutive games affected the psychological state of the players (31) and could be an explanation for the poor quality of players' sleep found by Cook and Charest (32), as fatigue increases with successive games, and other mood states such as tension, depression and anger may also do so as a function of the outcome of the games. However, future research is needed that analyzes the psychological state of players and the quality of players' sleep together, and may even include heart rate analysis, as previous research in traditional sports showed that consecutive games influenced heart rate variability (29), and previous research in esports confirms that the competition and the game situation can produce an activation of the sympathetic nervous system (13), which could affect rest.

In this regard, heart rate and heart rate variability have been used in recent years in the esports field for their potential to monitor player self-regulation (16). Thus, changes in the HRV time-domain variables during the games, as well as a decrease in the mean standard deviation of RR intervals have been observed in the winning teams compared to the losing teams, with some areas of analogous change in heart rate variability also existing in players at the end of matches (17). Similarly, heart rate showed significant differences depending on the play in which the players were involved, with those in which the player was directly involved and favored the team being the most decisive in these changes (7). All of this becomes even more relevant because previous research has suggested that the psychological states of players are related to certain physiological changes, including changes in heart rate (13).

In addition, another relevant aspect for esports performance is eye and cognitive fatigue. In this sense, it has been observed that both factors can be determinant for esports performance, decreasing it as fatigue increases. Although there is no specific research in the field of esports in which the psychological state is related to eye and cognitive fatigue, previous research in other areas can provide relevant information. Thus, in a study carried out with experienced drivers in a driving simulator to try to find out which aspects were most determinant for the onset of fatigue, it was shown that one of the associated factors was negative moods (58). In addition, the sympathetic and parasympathetic nervous systems have been shown to control the relationship between mental fatigue and tonic pupil size and have the potential to indicate mental fatigue (59). Therefore, although future research is required to analyze the most relevant factors in fatigue, mood states could be key in the appearance of visual and cognitive fatigue, as well as in the decrease of performance in esports.

With the results obtained in the present investigation we can partially reject H1 in which it was indicated that the mood of amateur LOL players will be affected by the outcome of each game, with the mood worsening to a greater extent with defeats occurring consecutively compared to defeats following a win. This is because fatigue increased significantly after the games, independently of the final result of the games. However, depression, anger and tension increased with the first defeat, but after the second defeat there was not an even more significant increase.

The results obtained allow the possible extrapolation of these results to the professional environment. Although the findings obtained should be corroborated in future research because the analysis of successive games had not been performed in previous scientific literature, neither in the professional nor amateur field, what is shown is that in successive games the mood of the players is modified by the result of this one, except fatigue that seems to increase regardless of the result. Therefore, the use of certain strategies or psychological interventions between the breaks of competitive games could be considered to readjust the state of mind, predisposing the athlete to face the next game in the best possible conditions. In addition, it seems that the variables on which to influence with psychological intervention could differ depending on the outcome of the game, since depression, anger and tension seemed to fluctuate the most when defeat occurred. The increase in the fatigue variable between successive games is also important because makes it necessary to consider the importance of offering rest to the players between each game played.

In addition, it is important to note that the mood of the players would be affected when they play LOL games. This is decisive, firstly, because defeat in a given game can be the origin of disruptive behaviors in the following games, referring to what is known as tilt in the field of esports (23). Secondly, this could have consequences on the well-being of the players, since subjective well-being is related to moods (60), which is especially relevant in young or vulnerable populations in which competitive video games, such as LOL, could have a negative effect due to the influence of the final result. However, these results should be corroborated in future research, in which it could be analyzed whether victory or defeat in other competitive esports can also affect the mood of players, as this would allow generic recommendations to be made for the population, with special emphasis on the effect that certain video games could have on the most vulnerable populations. Moreover, in LOL, future research should analyze how long it takes for moods that have been altered to return to baseline levels or what strategies can be really useful so that players do not see their games turned into negative gaming experiences.

Therefore, the practical implications of the results obtained in the present investigation are (1) the moods of the final moments of the game seem more relevant than those of the beginning; (2) the outcome of the previous game may be determinant in the mood with which players face the next game, and although the previous mood does not influence game performance, this could be relevant in terms of its effect on sleep, eye and cognitive fatigue, or the associated changes in heart rate. Therefore, the relevance of mood changes is not only due to their direct influence, but to the effect on other physiological variables.

The present study is not free of limitations. The sample was small and was selected by convenience, by choosing the subjects who wished to participate in the research. Regarding the intervention, it should be noted that the exclusive use of the POMS questionnaire provides relevant information, but it only addresses a small part of the psychological domain, so future research should analyze other psychological variables, as well as include other methods such as interviews to gather more information. In addition, other aspects should be considered in future research, since the composition of the team, the playing position, or other factors specific to the game could be determinant in the psychological state of the players. In addition, player mood was not related to players' perception of fatigue. Therefore, future research should assess the perception of fatigue or effort using scales such as the rate of perceived exertion (RPE), which can provide much information by comparing whether the effort that players have perceived to face the game corresponds to their mood, and whether when the effort is greater it affects mood to a greater extent. This becomes even more relevant in situations where several games are played in a row (Bo3 or Bo5) because the fatigue dragged from one game to another can reduce the psychological state with which the next game is faced. Although the games were ranked, the amateur environment lacks professionalism, which could diminish the importance that players attach to winning or losing a game, so future research should be conducted with professional players during competition as the psychological response may be totally different. And finally, the fitness condition of the players (such as the handgrip test), and its possible relationship with performance, was not assessed. Although previous research has shown virtually no relationship between fitness and esports performance (61), this may be because the tests included were limited, not fully assessing players' physical fitness, so future research could examine whether higher performance in certain upper body fitness tests (such as the handgrip strength test) is relevant to esports-specific performance. Taking into account the existing limitations, the present research is a first approximation to the effect that the results of consecutive games can have on the mood of LOL players, which provides very relevant information for the competitive and recreational environments.

## 5. Conclusions

The mood prior to the game did not seem to be determinant in the subsequent performance during the game. However, the mood of amateur LOL players was influenced by the outcome of the game (win or lose), with an increase in tension, depression and anger found when the game was lost. In addition, analysis of successive games showed that when the first game was lost and the second game was won, the increases in depression and anger found with the first loss were reduced with the subsequent win. However, fatigue was the only state of mind that increased after two consecutive victories, so that the rest time between games becomes very important, especially in amateur players where the time between games is not regulated. The relationship between mood states, well-being, heart rate, and cognitive and visual fatigue are discussed in this research, which gives greater importance to these results, since most amateur gamers use video games as a form of distraction and entertainment, but this aim could be diminished based on the results obtained.

## Data Availability

The raw data supporting the conclusions of this article will be made available by the authors, without undue reservation.
